# Central Auditory Processing Disorder in Children: A Multidisciplinary Approach

**DOI:** 10.7759/cureus.116936

**Published:** 2026-09-25

**Authors:** Mohammed K Alnoury, Jeanane M Ferre, Alyaa J Hemdi, Haythem Abuzinadah, Ibrahim Alnoury, Khaled I Alnoury

**Affiliations:** 1 Department of Otolaryngology - Head and Neck Surgery, Faculty of Medicine, King Abdulaziz University, Jeddah, SAU; 2 Audiologist, Private Practice, Oak Park, USA; 3 Department of Clinical Psychology, College of Applied Medical Sciences, King Abdulaziz University, Jeddah, SAU; 4 Department of Otolaryngology - Head and Neck Surgery, King Abdulaziz University Hospital, Jeddah, SAU

**Keywords:** capd, central, central auditory processing disorder, children, hearing, multidisciplinary

## Abstract

Central Auditory Processing Disorder (CAPD) is an impairment in the neural processing of sound information across the central auditory nerve system, not caused by peripheral hearing loss. The literature has highlighted the relationship between CAPD and learning difficulties and other conditions such as attention deficit hyperactivity disorder (ADHD). This comprehensive review aims to describe a multidisciplinary clinical approach utilizing perspectives from otolaryngologists, audiologists, and psychologists. An expert panel of otolaryngologists, audiologists, and psychologists reviewed the literature and leveraged their clinical expertise to outline the multidisciplinary approach. The otolaryngologist's role is to comprehensively examine the patient's medical history and head and neck region to identify any potential causes of hearing loss, such as genetic or acquired factors. The audiologist conducts specialized tests specifically designed to assess central auditory processing abilities. Additionally, psychological assessment plays an important role in understanding the impact of cognitive and emotional factors on CAPD. The multidisciplinary approach detailed in this study will assist clinicians in establishing multidisciplinary clinics that would facilitate accurate diagnoses and provide personalized interventions, such as speech and language therapy and accommodations in academic settings.

## Introduction and background

The term auditory processing refers, in general, to “what we do with what we hear,” encompassing an array of skills beginning at the outer ear and ending when a response is provided - even if the response is incorrect. Skills include signal detection at the peripheral ear and auditory nerve, analysis and synthesis of the acoustic signals in the central auditory nervous system, meaning attachment in the central language system, and execution of a response, subserved by the frontal lobes and motor cortex. An impairment in the neural processing of sound information across the central auditory nervous system that is not caused by peripheral hearing loss is referred to as central auditory processing disorder (CAPD) [1,2]. In everyday situations, children with CAPD may hear sounds normally but have difficulty making sense of spoken information, particularly when listening conditions are challenging. For example, a child may have difficulty following a teacher's instructions in a noisy classroom or understanding conversation when several people are speaking, despite having normal peripheral hearing. These difficulties reflect challenges in the way the brain processes and interprets auditory information rather than simply an inability to hear sounds. Central auditory skills can be influenced by attention, memory, listening demeanor, motivation, wellness, and cognition. Thus, functional issues in auditory processing, i.e., day-to-day listening and usage, can result from and/or co-exist with specific auditory impairment, language impairment, neurocognitive dysfunction, psychosocial disorders, or health issues [1,2]. Some children may be labelled as inattentive, forgetful, or delayed before it is recognised that they have CAPD. As a result, early detection of CAPD and rapid treatment will greatly benefit their auditory training. The general population is estimated to have a prevalence of CAPD ranging from 0.5% to 10%, while in children, the estimated prevalence is approximately 3% to 5% [3,4]. The assessment of CAPD can be challenging because auditory processing difficulties may coexist with or overlap with language, cognitive, attentional, developmental, and peripheral hearing disorders. Accordingly, the diagnosis of CAPD requires a comprehensive evaluation that incorporates standard audiological assessment alongside cognitive, memory, speech and language, behavioral, psychological, electrophysiological, and advanced auditory processing measures [5]. Therefore, a multidisciplinary approach is important: otolaryngology helps identify or exclude peripheral and medical causes of hearing difficulties, audiology evaluates auditory processing abilities using behavioral and physiological measures, and psychology and related disciplines assess cognitive, language, attention, and other factors that may influence auditory performance. Integrating these perspectives helps distinguish central auditory processing difficulties from other conditions that may present with similar listening or communication challenges. The objective of this review is to describe a multidisciplinary clinical approach to CAPD in children, considering the perspectives of otolaryngology, audiology, and psychology.

## Review

Methodology

This narrative review was developed through a multidisciplinary expert panel comprising specialists in otolaryngology, audiology, and psychology. The panel consisted of four otolaryngologists, an expert audiologist in CAPD, and a psychologist. Relevant literature was identified and selected based on its scientific and clinical relevance to the assessment and management of children with suspected CAPD.

The panel critically considered the available evidence within their respective areas of expertise and integrated these perspectives to develop a clinically applicable framework for the assessment and management of children with suspected CAPD. This approach was intended to provide a balanced synthesis of the current evidence while incorporating multidisciplinary clinical expertise rather than a systematic review or quantitative synthesis.

Otolaryngologist Assessment

Understanding the various causes of hearing loss in children is essential for effective diagnosis and management. Hearing loss can be classified according to the etiological factors, which include genetic/hereditary and non-genetic causes. The hereditary causes can be subcategorized into syndromic and non-syndromic causes. Hearing loss can also be classified based on the time of onset into congenital (present at birth) and acquired hearing loss (occurring after birth). Additionally, hearing loss can be classified anatomically into conductive, sensorineural, or mixed [6,7]. Based on these classifications, the multidisciplinary team can guide further workup and counselling.

The otolaryngologist evaluation starts with a thorough history focusing on known perinatal risk factors such as (1) prematurity, (2) length of intensive care unit (ICU) admission, (3) history of ventilation, (4) hypoxia, (5) sepsis, (6) hyperbilirubinemia, (7) exposure to ototoxic medications, and (8) history of intrauterine infections. Family history of hearing loss and consanguinity are reviewed, and inquiries into developmental milestones related to motor and language skills are conducted.

Additional questions are directed towards acquired causes of hearing loss such as (1) history of ear infections, (2) otorrhea, (3) vertigo, (4) imbalance, (5) tinnitus, (6) middle ear effusion, (7) trauma, (8) previous ear surgeries, (9) history of meningitis, and (10) noise exposure [7-11]. Further evaluation is done to identify conditions such as autism or attention-deficit/hyperactivity disorder (ADHD), given the existing evidence suggesting a connection between CAPD and these disorders [12,13].

General and head & neck examination: Children with hearing loss should undergo a general examination, including a through head and neck assessment, to check for syndrome-related disorders. These may include identifying features such as (1) white forelock, (2) dystopia canthorum, (3) heterochromic irides associated with Waardenburg, (4) preauricular pit and branchial anomalies associated with branchio-oto-renal (BOR), (5) blue sclera characteristic of osteogenesis imperfecta, (6) other craniofacial anomalies such as aural atresia and cleft palate, (7) goiter related to Pendred syndrome, and (8) other limbs and phalangeal defects [7-11]. These findings are linked with specific syndromes, and sensorineural hearing loss is typically a common feature among these cases.

Otoscopic and microscopic ear examination: An otoscopic and microscopic examination is conducted to rule out causes of conductive hearing loss such as (1) aural stenosis/atresia, (2) wax impaction, (3) signs of external or middle ear infections, (4) perforated tympanic membrane, (5) evidence of middle ear effusion, (6) retracted tympanic membrane, and (7) cholesteatoma.

Vestibular and balance assessment: An age-appropriate assessment of vestibular and balance function is recommended in children with sensorineural hearing loss. For children older than four years, this may include (1) single-leg standing with the eyes open and closed, and (2) tandem gait assessment with the eyes closed [11]. These behavioral assessments may be complemented by age-appropriate vestibular evoked myogenic potential (VEMP) testing.

Audiologist Assessment

Development of central auditory processing skills: The auditory skills that fall under this definition include (1) binaural interaction, which refers to the ability to integrate information received by both ears; (2) auditory discrimination, or the ability to distinguish between different sounds; (3) auditory pattern recognition, or the ability to recognize patterns in auditory information; (4) dichotic listening, which involves processing different auditory information presented simultaneously to each ear; and (5) interhemispheric integration, which refers to the coordination of auditory information between the two cerebral hemispheres [14].

From a neurodevelopmental perspective, these processes emerge at different times during childhood and are typically well established by approximately six years of age, allowing them to be assessed using age-appropriate measures. The development of these auditory abilities can also be viewed within Erber’s hierarchy of auditory skill development, which provides a general framework describing the progression from detection and discrimination to identification and comprehension. This hierarchical progression reflects the increasing complexity of auditory processing and the development of functional listening abilities during childhood [15-18].

Children aged six years and above can be diagnosed and assessed using a variety of behavioral central auditory processing tests. Children between four years and six years can be assessed using some of the formal tests as well as some informal measures or indices that can help to identify the preschool precursors of the auditory processing skills. These measures are not necessarily diagnostic in preschool age children; however, they can give insight to a child's auditory processing abilities and provide a basis for subsequent assessment. There are no behavioral tests available to assess central auditory processing in children less than four years of age [5,18,19].

The understanding of typical developmental characteristics of skills that are mediated by the peripheral and the central auditory systems will enable the clinician to assess auditory processing in children, to identify possible delays or disorders, and to predicate on that the functional impact that this will have on the listener of oral information. Deficit-specific intervention strategies will then be developed in order to tackle the child's weaknesses as effectively as possible and to prevent any possible negative effects [5,14].

Comprehensive audiological assessment: For a comprehensive evaluation of a child with a suspected CAPD, the audiological evaluation includes a series of complex tests which are intended to challenge the auditory system in various ways, that is to ‘overwork’ it. Central auditory tests are distinct from simple hearing tests as they attempt to determine how efficiently the auditory system uses the auditory information transmitted by the ear. Results of the child are compared to those of an age-matched peer group and a performance profile for the child is established to assist in defining the nature of their CAPD [5,14].

Factors affecting assessment and interpretation: Testing central auditory processes can be contraindicated or challenging for a number of individuals. The majority of currently available behavioral tests for measuring central auditory processes were designed for use with individuals who have normal to near-normal peripheral hearing. In addition, the language in which testing is administered is an important consideration, as auditory processing tasks may be influenced by the linguistic background of both the patient and the test materials. Whenever possible, assessments should be administered using tests available in the patient’s first or most proficient language, with appropriate consideration of the language background of the examiner. Automated or language-independent measures may help address this challenge in some settings.

However, the limited availability of validated central auditory processing tests across different languages remains an important limitation in the assessment of CAPD, particularly in multilingual populations. For clients with marked hearing loss in the better ear, test results may be confounded by a number of factors and, consequently, interpretated incorrectly. Many current behavioral tests of central auditory processing require a verbal response, typically in the form of an oral speech response. Even though a number of the central auditory tests have a picture-pointing alternative, it is important to realize that such testing also includes visual aspects that can influence test results. In addition, the vocabulary used in a number of the diagnostic tests of central auditory processing is such that it is generally not appropriate for use with children younger than four years of age and adolescents and adults with language deficits of varying degrees. The majority of current tests of central auditory processing have normative data for individuals of average intelligence. Thus, results obtained from individuals of below average intelligence as well as those of below normal intelligence should be interpreted with great caution and always in relation to the client's overall cognitive abilities as well as their linguistic abilities [20].

Diagnostic test battery and interpretation: The use of a test battery for differential diagnosis of CAPDs is endorsed by most audiologists [5,21]. Although there is less agreement concerning the test(s) that should be part of the test battery for the identification of CAPDs, a comprehensive diagnostic approach would include a variety of behavioral measures and electrophysiologic/electroacoustic tests. A diagnostic central auditory evaluation typically includes the following elements: (1) pure-tone air & bone conduction audiometry, often otoacoustic emission tests, (2) speech reception threshold and word recognition tests, (3) immittance audiometry (tympanometry and acoustic reflexes), (4) binaural interaction tasks such as speech-in-noise to examine brainstem-based skills, (5) monaural low redundancy (degraded) speech tests to tax auditory discrimination skills, (6) dichotic listening tests that are sensitive to deficits in cortical binaural integration and separation skills, and (7) tonal patterning tests that tax right hemisphere-based pattern recognition as well as interhemispheric integration skills [5,14,22,23]. 

Performance >2 standard deviations (SDs) below the mean for age is considered abnormal for any individual diagnostic test. Performance below normal limits on a single central auditory test is not considered diagnostically significant for specific central auditory deficit. Conversely, failure on all tests does not indicate specific central auditory impairment. Evidence of specific central auditory processing disorder is defined as poor performance on sets of tests taxing similar skills [24]. The type of disorder, if present, is revealed by examining patterns of performance across all test scores, both normal and abnormal. Results provide information regarding peripheral auditory function and central auditory processes including binaural and temporal processing and auditory discrimination.

Management of central auditory processing disorder: Once a diagnosis of specific central auditory processing disorder is made, an intervention plan of deficit-specific treatment, environmental modifications and compensatory strategies can be developed, which is designed to reduce/resolve the auditory deficit and minimize its impact on the listener’s life [25,26]. Environmental modifications are designed to improve signal clarity and access [27] and include noise abatement, preferential seating, use of Clear Speech [28] and use of assistive listening technology [29]. Compensatory strategies help mitigate the functional impact of auditory impairment on communication and learning skills and include extended time for tests and/or projects, modification of curricular and/or workplace requirements, and access to assistive computer technology [27,30]. Direct remediation, i.e., therapy improves deficient skills and teaches compensatory/coping strategies [26].

Auditory training and treatment outcomes: A wide variety of options exist for auditory training, including computer-based programs, designed to improve specific central auditory processing skills and related phonologic, linguistic, and visual skills of children and adults [31,32]. Treatment outcome data, to date, provide support for the use of auditory training, including computer-based programs in reducing/resolving specific central auditory impairment among children and adults [33-36]. However, the available evidence is heterogeneous in terms of study design, outcome measures, and methodological quality, and these findings should therefore be interpreted with appropriate caution.

Table 1 and Figure 1 describe a proposed clinical approach and pathway for suspected CAPD in children.

**Table 1:** Proposed clinical approach to the audiological assessment and management of children with suspected CAPD CAPD: Central Auditory Processing Disorder; SD: Standard Deviation.

Stage	Age / Population	Recommended approach	Key considerations
1. Developmental consideration	<4 years	Developmental surveillance and assessment of auditory behaviors	No validated behavioral CAPD tests are currently available; assess developmental auditory, speech, language, and communication skills.
2. Early assessment	4–6 years	Selected formal auditory processing measures supplemented by informal measures/indices	Results should generally be considered descriptive rather than definitively diagnostic; establish a baseline for later assessment.
3. Formal CAPD assessment	≥6 years	Comprehensive behavioral central auditory test battery	Consider age-appropriate normative data and the child's cognitive, linguistic, and developmental abilities.
4. Peripheral auditory evaluation	All ages, as appropriate	Pure-tone audiometry, speech testing, tympanometry, acoustic reflexes, and otoacoustic emissions when indicated	Establish peripheral auditory status before interpreting central auditory test results.
5. Central auditory assessment	Primarily ≥6 years	Assess binaural interaction, auditory discrimination, dichotic listening, temporal/pattern recognition, and interhemispheric processing	Use multiple tests targeting related auditory processes rather than relying on a single test.
6. Interpretation	All assessed children	Evaluate the overall pattern of performance across the test battery	An isolated abnormal test should not be considered diagnostic of a specific CAPD; performance >2 SD below age-based norms is generally considered abnormal.
7. Differential diagnosis	All assessed children	Integrate auditory findings with speech-language, cognitive, developmental, and educational information	Consider alternative or coexisting conditions that may influence test performance.
8. Individualized intervention	Children with identified deficits	Deficit-specific auditory training, environmental modifications, and compensatory strategies	Intervention should be based on the child's specific auditory difficulties and functional needs.
9. Follow-up	After intervention	Monitor functional outcomes and reassess auditory skills when clinically indicated	Evaluate improvement in auditory performance and, importantly, communication and academic functioning.

**Figure 1:**
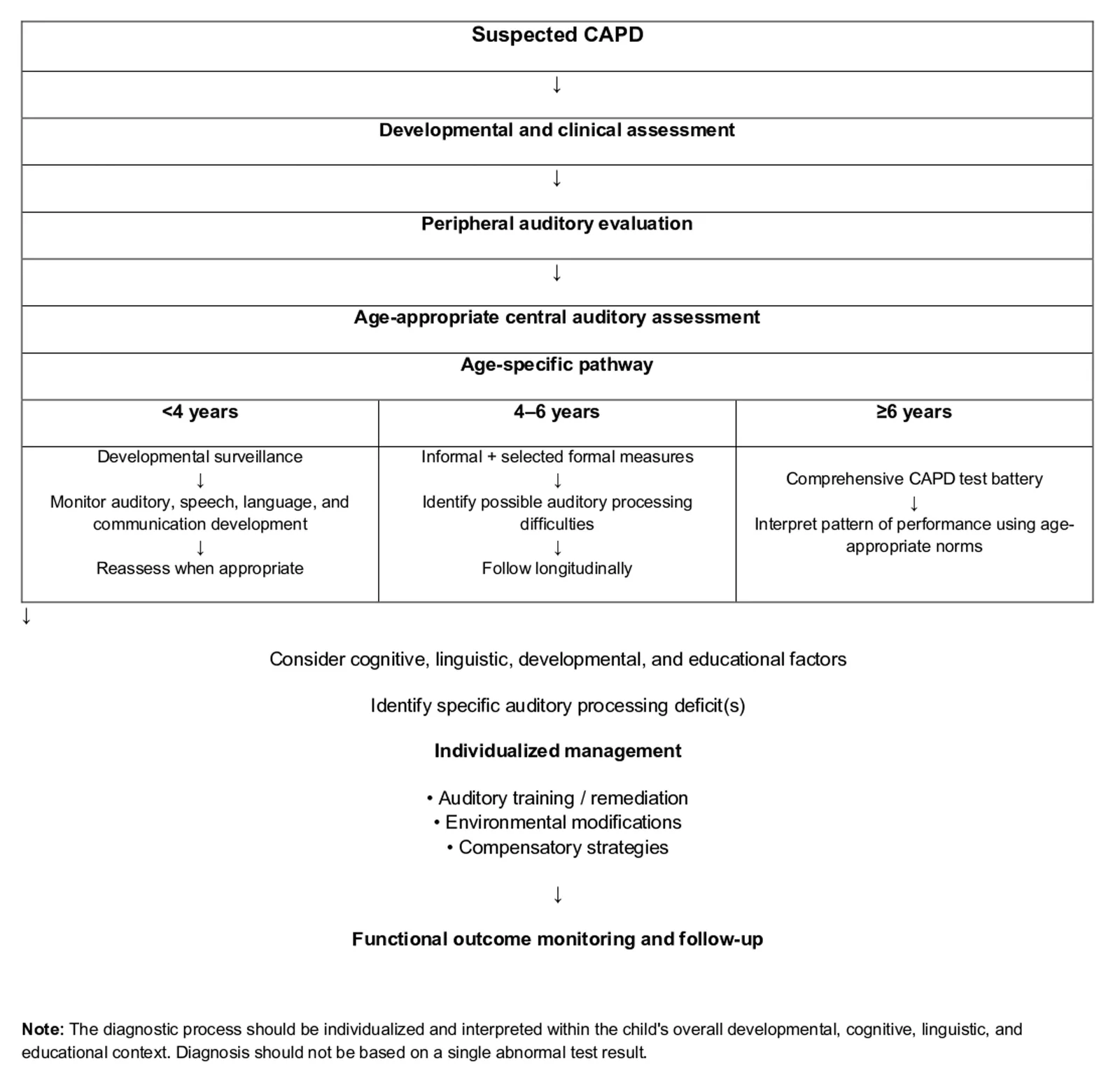
Proposed audiological pathway for children with suspected CAPD CAPD: Central Auditory Processing Disorder.

Psychological Assessment

The psychological assessment informs the team about the cognitive and emotional aspects related to CAPD, which helps to guide interventions that address not just the auditory processing challenges but also associated behavioral and learning difficulties [37]. This assessment involves a combination of neuropsychological evaluations to measure cognitive skills associated with auditory processing [30].

Processing tests: These evaluations assess an individual’s ability to process and comprehend speech as well as other sounds. Tests such as the Test of Auditory Processing in Children (TAPS-3) and Tests for Auditory Processing Disorders for Children (Third Edition) SCAN-3: C are commonly used for this purpose [38,39].

Language tests: These assessments evaluate an individual’s proficiency in understanding and utilizing language effectively. Common language tests used in this regard are the Clinical Evaluation of Language Fundamentals (CELF) and The Test of Early Language Development, Third Edition (TELD-3) [40,41].

Attention and memory tests: These evaluations assess an individual’s capacity to pay attention to and process information efficiently. Examples of tests include the Test of Variables of Attention (TVA) and the Wechsler Memory Scale - fourth edition (WMS-4) [42,43].

Intelligence tests: These assessments measure cognitive abilities that may influence auditory processing, such as processing speed, working memory, and executive functions. Examples of these tests are the Wechsler Intelligence Scale for Children, Fourth Edition (WISC IV) and The Kaufman Brief Intelligence Test-second edition (KBIT-2) [44,45].

Behavioral observations: The evaluator will examine the individual's behaviour while performing tasks and having conversations. This observation aids in identifying any delays in response time, difficulties following directions, or misinterpretations of sounds [46]. The Children's Auditory Performance Scale (CHAPS) is an example of an observational scale utilised in this case, which includes questions concerning the child's auditory behaviour in a variety of settings such as home, school, and public places [46]. It evaluates hearing in noise, following directions, and auditory attention. Another example is The Fisher's Auditory Problems Checklist, which is intended to identify specific auditory processing abnormalities [47]. It contains questions about sound localization, auditory discrimination, and interpreting speech in noisy environment.

Following the assessment, a multidisciplinary team of otolaryngologists, audiologists, and psychologists thoroughly reviews and analyses medical, audiological, behavioural observations, and neuropsychological evaluation data to determine whether the person meets the diagnostic criteria for CAPD [48]. The responses may show patterns typical with CAPD, such as difficulties processing speech in noisy situations or trouble following multi-step instructions [49]. This aids in distinguishing CAPD from other illnesses, as some patterns are more typical of CAPD. Based on the results of the evaluation, recommendations will be made to help individuals with their issues with processing information. These recommendations may involve speech and language therapy accommodations at school, work, or programs designed to enhance processing skills [37]. Moreover, it is essential to consider CAPD prevalence in children with learning difficulties and its association with attention deficit hyperactivity disorder (ADHD) for targeted interventions [50-52].

There are no specific cognitive battery tests for CAPD making the diagnosis more challenging, particularly given that ADHD presents with CAPD-like symptoms, adding more challenges [53]. In addition, language and cultural barriers may significantly affect testing and diagnosis, for example, children in non-English speaking homes may struggle with English testing, which can lead to disabilities [53]. These findings underscore the need of addressing the challenges and limitations involved with conducting psychological evaluations for CAPD in order to provide an accurate diagnosis and suitable care.

Limitations

This review is not a systematic review; therefore, a predefined systematic search strategy was not undertaken to identify all potentially relevant studies, and the included evidence was not formally evaluated using a standardized quality assessment instrument. Consequently, the findings should be interpreted in the context of the narrative nature of the review and the available evidence.

Strengths

The principal strength of this review is its comprehensive, multidisciplinary approach to the assessment and management of children with suspected CAPD. Perspectives from otolaryngology, audiology, and psychology are integrated to provide a practical framework that may assist clinicians in establishing or enhancing multidisciplinary assessment services for these children. The review emphasizes that the diagnosis of CAPD should not be based on a single test or isolated finding; rather, it requires interpretation of performance patterns across a comprehensive battery of behavioral and/or electrophysiologic /electroacoustic measures. In addition, the review considers potential confounding factors, including peripheral hearing loss, language delay, cognitive deficits, attentional difficulties, and developmental delay. It also addresses important age-related and test-related considerations relevant to the assessment of younger children, particularly those with language or cognitive difficulties. Collectively, these features enhance the clinical applicability of the review and may provide a useful resource for clinicians involved in the evaluation and management of children with suspected CAPD.

## Conclusions

A multidisciplinary approach combining otolaryngologists, audiologists, and psychologists enables a comprehensive assessment and personalized management strategy for children with CAPD. Specialized auditory test batteries provide valuable information regarding auditory processing abilities and help identify patterns of CAPD-related deficits. However, interpretation of auditory test results should be integrated with assessments of cognitive, language, attention, and behavioral factors, which may influence auditory performance and contribute to overlapping clinical presentations. This comprehensive approach can help distinguish CAPD from other conditions with similar manifestations, including learning difficulties and ADHD. Early recognition of individual strengths and weaknesses can facilitate the development of targeted interventions, environmental modifications, and compensatory strategies tailored to each child's needs. Overall, a coordinated multidisciplinary framework may support more accurate clinical assessment and individualized management while minimizing the functional impact of auditory processing difficulties on children's communication, learning, and daily activities.
